# Reducing Dietary Protein Enhances the Antitumor Effects of Chemotherapy through Immune-Mediated Mechanisms

**DOI:** 10.1158/1535-7163.MCT-24-0545

**Published:** 2025-07-02

**Authors:** Samantha C. Mulkeen, Suchandrima Saha, Carmen R. Ferrara, Vladimira Bibeva, Michael C. Wood, Ji Dong K Bai, Tanara V. Peres, Daniel Martinez-Martinez, Alex Montoya, Pavel Shliaha, Filipe Cabreiro, David C. Montrose

**Affiliations:** 1 Department of Pathology, Renaissance School of Medicine, Stony Brook University, Stony Brook, NY, USA; 2 Institute of Clinical Sciences, Imperial College London, London, UK; 3 CECAD Research Cluster, University of Cologne, Cologne, Germany; 4 Stony Brook Cancer Center, Stony Brook, NY, USA

**Keywords:** proteomics, colon cancer, unfolded protein response, amino acids

## Abstract

Diet is believed to be an important mediator of oncogenesis and response to anti-cancer therapies, although no evidence-based dietary guidelines exist for patients with cancer. Limiting protein intake can suppress tumor growth by both inducing nutrient stress and enhancing anti-tumor immunity. However, little is known about the impact of reducing dietary protein on the efficacy of chemotherapy, the most widely used anti-cancer treatment. Here, we present evidence that reducing protein intake in mice by 50% stops the growth of established tumors, in parallel with inducing a stress response and DNA damage. Further, a reduced protein (RP) diet enhances tumor regression upon treatment with 5-fluorouracil (5-FU). This effect is accompanied by elevated apoptosis and suppressed mitosis of tumor cells. Proteomic analysis of tumors revealed marked differences between 5-FU treated mice fed control or RP diet including decreased abundance of proteins that mediate DNA repair and replication in mice consuming RP. *In vitro* studies mimicking amino acid changes found in tumors from RP-fed mice showed that cGAS/STING1 signaling, including transcription of *Interferon beta 1*, was maximally increased in 5-FU treated cells cultured in modified amino acid medium. These findings correlated with enhanced immune cell influx into tumors from mice treated with 5-FU while consuming a RP diet, an effect that was causally linked to improved response to chemotherapy. Collectively, these findings suggest that reducing dietary protein in cancer patients may enhance the efficacy of chemotherapy by promoting anti-tumor immunity.

## Introduction

The enhanced metabolic activity of cancer cells drives their avidity for nutrients to support pro-proliferative pathways. As such, limiting nutrient availability, including amino acids, can exert anti-cancer effects including induction of DNA damage and decreased nucleotide production in tumor cells (1–4). Acute stress induced by this nutrient deprivation can trigger the unfolded protein response (UPR) in the endoplasmic reticulum (ER), a signaling pathway that blocks protein translation, and in turn, prevents further accumulation of unfolded proteins as a pro-survival mechanism (5). Commonly used anti-neoplastic therapies, including chemotherapy, can induce cancer cell stress similar to nutrient deprivation, thereby supporting the concept of combining nutrient restriction with such treatments to enhance therapeutic response. In fact, previously conducted studies have shown that depriving select amino acids or other nutrients, while simultaneously treating tumors with chemotherapy or immunotherapy, enhances the therapeutic response in preclinical cancer models (4,6–8). However, the optimal clinically-actionable approach to deprive tumors of growth-promoting nutrients in order to improve therapeutic efficacy, has yet to be determined.

Diet is believed to contribute to cancer development, progression, response to therapy, and ultimately patient outcome. For example, studies have shown that high protein intake significantly increases the likelihood of dying from cancer, and work in rodents demonstrates that feeding a high protein diet enhances hepatic carcinogenesis (9–11). Amino acids, the components of protein, are critical mediators of cancer cell metabolism, and as such, support tumor growth and mediate resistance to anti-cancer therapy (6,12,13). Moreover, they contribute to the composition of the tumor microenvironment (7). Given these roles, extensive work has been carried out to inhibit amino acid synthesis and/or uptake (including through diet) as anti-neoplastic strategies (1,14). However, blocking generation or transport of select amino acids is challenging. As a potential method to overcome this issue, reducing dietary protein has been proposed as a method to globally reduce amino acid availability. In fact, feeding a reduced protein diet to mice suppresses tumor growth, an effect mediated through both cancer cell intrinsic mechanisms (e.g. reducing insulin-like growth factor 1 signaling and inhibiting mTOR activity), as well as enhancing the anti-tumor immune response (15–18). Notably, both of these mechanisms appear to be causally linked to nutrient deprivation-driven tumor cell stress (15–18). However, the impact of reducing dietary protein on the anti-tumor effects of chemotherapy is less well understood.

The current study was conducted to determine whether reducing protein intake in mice enhances the therapeutic efficacy of the commonly used chemotherapy agent 5-fluorouracil (5-FU), and underlying mechanisms. Here, we demonstrate that feeding a reduced protein (RP) diet halts the growth of established tumors, in parallel with increasing the UPR, ROS levels and DNA damage. 5-FU treatment during RP diet feeding results in markedly enhanced tumor regression compared to a control diet, accompanied by alterations in the tumor proteome. These augmented anti-tumor effects were not cancer cell intrinsic, but instead activated the cGAS/STING1 pathway and enhanced infiltration of tumor-killing immune cells. Collectively, these findings support the concept that reducing protein intake may provide a beneficial response during chemotherapy treatment.

## Materials and Methods

### *In vivo* studies

All experiments involving mice were approved by the Institutional Animal Care and Use Committee of Stony Brook University (protocol # 1345543). For allograft studies of tumor growth, male BALB/c mice (RRID:IMSR_JAX:000651) were obtained from the Jackson Laboratory at seven weeks of age. Mice were anaesthetized using isoflurane and injected subcutaneously with 5 × 10^5^ of CT26 murine colorectal cancer cells (RRID:CVCL_7254) (kindly provided by L.A. Martinez of Stony Brook University in 2021) in a 1:1 ratio of Matrigel and serum-free RPMI 1640. Mice were fed AIN-93G diet (Research Diets) until tumors reached a palpable size of 200-250 mm^3^, after which, they were either continued on control (CL) or given a 50% reduced protein diet (RP) (Research Diets) (Table S1). Soybean oil was added to the RP diet to make it isocaloric with CL (Table S1). Select groups of mice also received 75 mg/kg of 5-fluorouracil (5-FU) (Sigma) dissolved in 0.9% saline, through intraperitoneal injections. Tumor volume was measured using skin calipers. For experiments using antibodies blocking CD4^+^ and CD8^+^ T cells, each tumor-bearing mouse received 400 μg of anti-CD4 antibody (BioXCell, clone: GK1.5; RRID:AB_1107636) and 200 μg of anti-CD8 antibody (BioXCell, clone 2.43; RRID:AB_1125541) or isotype control (Rat IgG2b, BioXCell, clone: LTF2; RRID:AB_1107780), by intraperitoneal injection on days 8 and 16 after CT26 cell inoculation.

For the orthotopic breast cancer model, 1 × 10^5^ 4T1 murine mammary tumor cells (obtained from ATCC in 2022) (RRID:CVCL_0125) were suspended in a mixture of 1:1 serum-free RPMI and matrigel and inoculated into the fourth mammary fat-pad. Tumors were allowed to grow until they reached a size of 200-250 mm^3^ while being fed AIN-93G. Mice were then randomized to receive either CL or RP diet while all mice received intraperitoneal injections of 5 mg/kg of doxorubicin (Dox) dissolved in 0.09% saline. To assess muscle mass the gastrocnemius muscle was dissected and either weighed or the length of the cross section was determined from images of the entire muscle using NIH ImageJ (version 1.53k).

### Cell culture

CT26 murine colon cancer cells and 4T1 murine mammary cancer cells were cultured in RPMI 1640 medium (Gibco) at 37 °C under 5% CO_2_ containing 10% fetal bovine serum (FBS) (Gibco) and 1% 100 U/mL penicillin sodium and 100 μg/mL streptomycin sulfate (Gibco). The cell lines were authenticated using short tandem repeat profiling (ATCC) on January 9, 2024 and all experiments were performed within six months of analysis. All the cell lines were routinely checked for mycoplasma using MycoProbe Mycoplasma Detection Kit (R&D Systems) and all experimentation was performed within the following month. All cells were used within 10 passages for experiments. Control medium for experiments was generated using Dulbecco’s Modified Eagle Medium F-12 powder (USBiological Life Sciences) and supplemented with 10% dialyzed FBS (Gibco), 1% 100 U/mL penicillin sodium and 100 μg/mL streptomycin sulfate, 15 mM HEPES, 5mM glucose, 0.05 mM sodium pyruvate and 23.81 mM sodium bicarbonate. Amino acids (Sigma-Aldrich) were added at concentrations that mimic human plasma-like medium (HPLM, Thermo Fisher Scientific). Modified amino acid medium (Modified AA) contained all components except the concentrations of amino acids were adjusted based on the fold changes of amino acid levels in tumors from mice fed control or 50% protein diet (see Results section). Each medium was balanced to pH 7.4 and sterile filtered using a 0.2 μm filter. Complete formulations of control and Modified AA media are shown in Table S2.

### Cell viability assay

To determine cell viability, 1 x 10^5^ CT26 cells were seeded on 12-well plates and given control or Modified AA media and treated with vehicle or 10 μM of 5-FU for 72 h. Cells were then harvested and stained for 30 minutes at 37°C with 20 nmol/L of 3, 30-Dihexyloxacarbocyanine iodide (DiOC6) (Sigma-Aldrich) followed by staining with 1 μg/mL of 40,6-diamidino-2-phenylindole (DAPI) (Sigma-Aldrich) for 5 minutes. The samples were then subjected to flow cytometric analysis using a BD LSRFortressa II flow cytometer with 405 nm (violet) and 488 nm (blue) lasers. DIOC6 positive and DAPI negative cells were considered live. Data were analyzed using BD FACSDiva 9.0 software.

### Cell proliferation assay

Cell proliferation was assessed by determining the cell count based on CountBright absolute counting beads (Thermo Fisher Scientific). Briefly, 1 x 10^5^ CT26 cells were seeded into 6-well plates and serum starved for 18 h. Cells were then incubated with control or Modified AA media for the indicated number of days. On the day of analysis, cells were harvested, stained with DiOC6 and DAPI, and 50 mL of counting beads were added. The samples were acquired using 640 nm (red) laser excitation on BD LSRFortressa II flow cytometer (BD). Cell concentration was determined using the formula provided by the manufacturer and the number of cells on each day was calculated as relative to the number of cells on day 0. Data were analyzed using BD FACSDiva 9.0 software.

### ROS measurements

CT26 cells were seeded at a density of 3 × 10^5^ in 12-well plates and were given either Control or Modified AA media for 12 hours. Cells cultured in control medium received vehicle (PBS) and cells given Modified AA medium were treated with either PBS or N-Acetylcysteine (NAC). Cells were harvested, washed with PBS and then incubated for 30 min at 37°C with CellROX green (Invitrogen) at a final concentration of 5 μM in serum-free DMEM. Cells were washed twice with PBS and immediately resuspended in ice-cold PBS and counterstained with 1 μg mL^-1^ DAPI. Samples were acquired with 405 nm (violet) and 488 nm (blue) laser on an LSRII flow cytometer (BD) and analyzed using BD FACSDiva 9.0 software.

### Quantitative real-time PCR (qRT-PCR)

Total RNA was isolated using QIAzol (Qiagen) from CT26 cells subjected to control or Modified AA media for 6 h and then switched to RPMI 1640 medium for 18 h while treated with PBS, 2 μM, or 10 μM of 5-FU diluted in PBS. RNA quantity was determined using a NanoDrop ND-1000 spectrophotometer and cDNA was synthesized using 2000 ng total RNA using oligo (dT) primers and reverse transcriptase (Quanta). Quantitative real-time PCR was performed on real-time PCR detection system using PowerTrack SYBR Green PCR Master Mix (Thermo Fisher Scientific) and gene-specific primers for mouse *Ifnb1* (fwd: 5’-AAGATCAACCTCACCTACAG-3’; rev: 5’-AAAGGCAGTGTAACTCTTCT-3’). Mouse *Actb* (fwd: 5’-GGCTGTATTCCCCTCCATCG-3’; rev: 5’-CCAGTTGGTAACAATGCCATGT-3’) was used as a housekeeping gene. cDNA was amplified on the QuantStudio 7 Real-Time PCR System (Applied Biosystems) and relative expression was calculated using the ΔΔCt method.

### Immunoblotting

Tumor tissues were immediately stored at −80°C after harvesting, unless otherwise noted. Tumor and cell protein lysates were obtained using RIPA Buffer supplemented with 1 protease and phosphatase inhibitor tablet (ThermoFisher Scientific) per 10 mL, followed by sonication for 10 min and centrifugation at 15,000 rpm for 15 min at 4 °C. Protein concentration was determined in the supernatant using the DC Protein Assay Reagent (Biorad) and equal amounts of protein were resolved on 4-20% Criterion TGX Precast Midi protein gradient gels (Biorad). Proteins were then transferred to nitrocellulose membranes, blocked with 5% BSA in Tris-buffered saline with 0.1% Tween 20 (TBST) for 1 h at room temperature and incubated overnight at 4°C with primary antibodies targeting CHOP 1:2000 (Cell Signaling Technology; 2895T; RRID:AB_2089254), XBP1 and sXBP1 (1:2000) (Abcam; ab37152; RRID:AB_778939), Phospho-Histone H2AX (1:2000) (Cell Signaling Technology; 2577L; RRID:AB_2118010), Mn SOD (1:2000) (Enzo; ADI-SOD-110-D; RRID:AB_2039585), phospho-TBK1 (1:1000) (Cell Signaling; 5483; RRID:AB_10693472), TBK1 (1:1000) (Cell Signaling; 3504; RRID:AB_2255663), phospho-STING1 (1:1000) (Cell Signaling; 19781; RRID:AB_2737062), STING1 (1:1000) (Cell Signaling; 13647; RRID:AB_2732796) phospho-IRF3 (1:1000) (Cell Signaling; 29047; RRID:AB_2773013), IRF3 (1:1000) (Cell Signaling; 4302; RRID:AB_1904036), phospho-P70 S6 Kinase (1:1000) (Cell Signaling; 9205; RRID:AB_2734746) or anti-beta-Actin peroxidase (1:20,000) (Sigma-Aldrich; A3854). The blots were washed four times with TBST (5 min each) and incubated with horseradish peroxidase-conjugated anti-rabbit (1:3000) (Sigma; A0545; RRID:AB_257896) or anti-mouse (1:3000) (Sigma; A0168; RRID:AB_257867) secondary antibodies for 1 h at room temperature. The blots were washed four times for 5 min with TBST and bands were visualized using Clarity Max Western ECL Substrate (Bio-Rad) on a C600 Gel Doc and Western Imaging System operated by cSeries capture v.1.6.8.1110 (Azure Biosystems). Images of uncropped immunoblots are shown in Fig. S1.

### Immune cell profiling

Tumors were excised, washed with ice-cold PBS, weighed and minced into 1-2 mm^3^ pieces. They were then placed into a digestion solution comprised of RPMI 1640 containing 0.5 mg mL-1 collagenase D (Worthington Biochemical Corporation), 0.5 mg mL-1 dispase (Gibco) and 0.01 mg mL-1 DNase I (Sigma-Aldrich) and incubated for 30 min at 37°C while shaking at 220 rpm. The samples were vortexed and strained through a 70 μm cell strainer. Spleens were isolated, washed, weighed, placed onto 40 μm cell strainer and crushed using the flat end of a 1 mL syringe plunger. The tumor and splenic flow-throughs were centrifuged at 1000 g for 5 min at 4°C and the pellets were washed with PBS and incubated with ACK lysis buffer for 5 min at room temperature to lyse the red blood cells. Samples were then washed with FACS buffer (PBS + 2% FBS) followed by blocking of non-specific binding using 1:200 anti-Fc monoclonal antibody (Biolegend) for 15 min at room temperature. Live/dead staining was carried out using LIVE/DEAD Fixable Aqua Dead Cell Stain (Invitrogen) at a dilution of 1:500, and fluorochrome-conjugated antibodies specific to cell surface markers were used at a dilution of 1:200, as follows: CD45 (Biolegend, clone: 30-F11; RRID:AB_2565884), CD3ε (Biolegend, clone: 145-2C11; RRID:AB_469572), CD4 (eBiosciences, clone: RM4.5; RRID:AB_389303), CD8 (Biolegend, clone: 53-6.7; RRID:AB_312746), MHC class II (Biolegend, clone: M5/114.15.2; RRID:AB_2565976), CD11c (Biolegend, clone: N418; RRID:AB_830649), CD80 (Biolegend, clone: 16-10A1; RRID:AB_11126141) and CD25 (Biolegend, clone: PC61; RRID:AB_2564124). For intracellular staining, cells were stained with the cell surface markers as described above, fixed with 4% PFA in PBS for 20 mins, permeabilized with 0.5% Tween-20 for 5 mins, blocked using 2% FBS diluted in permeabilization buffer for 15 mins and then stained with fluorophore tagged antibodies at a dilution of 1:200 as follows: IFN-γ (Biolegend, clone: XMG1.2; RRID:AB_2563105), and Granzyme B (Biolegend, clone: QA16A02; RRID:AB_2728389). Samples were acquired on an LSRII flow cytometer (BD) and data were analyzed using the Kalluza C software (version1.1). Gating strategies to identify dendritic and T cell populations are shown in Figs. S2 and S3.

### Amino acid measurements

Analysis of amino acid levels in CT26 tumors was carried out at the Weill Cornell Medicine Proteomics and Metabolomics Core Facility. Metabolites were extracted by bead beating tissue in precooled 80% methanol, followed by incubation at -80° C for 4 h. Samples were then centrifuged at 12,000 × *g* for 20 min at 4°C. The supernatants underwent vacuum centrifugation, and the remaining material was re-suspended in mobile phase. Targeted LC-MS analyses were performed on a Q Exactive Orbitrap mass spectrometer (Thermo Scientific) coupled to a Vanquish UPLC system (Thermo Scientific). Separation of metabolites was carried out using a Sequant ZIC-HILIC column (2.1 mm i.d. × 150 mm, Merck). Mobile A consisted of 100% acetonitrile and mobile B consisted of 0.1% NH4OH/20 mmol/L CH3COONH4 in water. The gradient ran from 85% to 30% A in 20 minutes followed by a wash with 30% A and re-equilibration at 85% A. Relative quantification was performed based on the peak area for each amino acid. All data analysis was done using in-house written scripts and metabolite abundance was normalized to tissue protein concentration.

### Histologic analysis

Formalin-fixed, paraffin-embedded (FFPE) CT26 tumor tissues were processed, sectioned into 5 μm sections and stained with hematoxylin and eosin. The number of apoptotic bodies (using a previously published method (19)) and mitotic figures per 10 high power fields (40X magnification, corresponding to a 1.96 mm^2^ area) were quantified in the most cellular and mitotically-active area of each tumor, while avoiding areas of necrosis. Quantification was performed in a blinded fashion by a board-certified pathologist (J.D.K.B).

### Proteomics

Tumor samples were extracted with a Surfactant Cocktail-Aided Extraction/Precipitation/On-Pellet Digestion (SEPOD) by adding 3.5 μl of buffer for every mg of tissue and beating the pellet with a metallic bead for one hour at 70° C (20). Protein concentration was estimated by the Bicinchoninic acid assay (BCA) and adjusted to 2 g/L. Samples (40 μg) were reduced and alkylated by adding equal volume of water containing 20 mM Tris(2-carboxyethyl)phosphine (TCEP) and 40mM chloracetamide. The samples were subjected to precipitated assisted capture (PAC) by addition of 200 μg of hydroxyl beads (MR-HYX010) and 60 μl ethanol (21). The precipitated beads were washed three times with 80% ethanol and digested overnight in 40 μl of 50 mM ammonium bicarbonate buffer with 20 ng/ul trypsin and 10 ng/μl lysC while shaking at 1700 rpm in PCR plates. The digest was transferred to a new plate and the resin was washed with 10 μl of 1% trifluoroacetic acid (TFA) and the washing solution was combined with the digest.

Chromatographic separation was performed using an Ultimate 3000 RSLC nano liquid chromatography system (Thermo Scientific) coupled to a Q Exactive HF-X mass spectrometer (Thermo Scientific) *via* an EASY-Spray source. Electro-spray nebulisation was achieved by interfacing to Bruker PepSep emitters (PN: PSFSELJ20, 20 μm). Peptide solutions were injected directly onto the analytical column (self-packed column, CSH C18 1.7 μm beads, 300 μm × 35 cm) at a working flow rate of 5 μL/min for 4 minutes. Peptides were then separated using a 66 minute stepped gradient: 0-45% of buffer B for 66 minutes (composition of buffer A – 95/5%: H_2_O/DMSO + 0.1% FA, buffer B – 75/20/5% MeCN/H_2_O/DMSO + 0.1% FA), followed by column conditioning and equilibration. Eluted peptides were analysed by a mass spectrometer in positive polarity using a data-independent acquisition (DIA) mode as follows: an initial MS1 scan was carried out at 120,000 resolution with an automatic gain control (AGC) target of 3e6 for a maximum IT of 200 ms, m/z range: 350-1650. This was followed by 30 DIA scans with variable window width at 30,000 resolution. AGC target set to 3e6 with maximum IT on auto. Normalized collision energy was set to 27%. Total run acquisition time was 82 minutes.

Proteomic data were processed using the Spectronaut software platform (Biognosys, v16.3.221108.53000) (22). Analysis was carried out in direct DIA mode as follows: 1) *Pulsar Search*: library generation and database search were carried out using default settings for a tryspin/p specific digest as follows - missed cleavage rate set to 3 and variable modifications allowed for methionine oxidation and protein N-terminal acetylation. PSM, Peptide and Protein group FDR = 0.01. Searches were carried out against the Uniprot *Mus musculus* 1 gene per protein sequence database (downloaded 20/07/2022, 21,992 entries). 2) *Direct DIA analysis*: a mutated decoy database approach was employed with protein q-value cut-off for the experiment set to 0.01 at the identification level. Quantification set to MS2 with proteotypicity filter set to only protein group specific with no value imputation strategy employed. Protein quantification method set to MaxLFQ (23). Normalization strategy left at the default setting of local normalization. Data are available *via* ProteomeXchange with identifier PXD052110.

### Statistical analysis

Cell proliferation and tumor growth rates were compared using F-test for equality of regression slopes. Significant differences in the number of apoptotic bodies, mitotic figures, gene expression and immune cell abundance were determined by unpaired two-tailed t-tests and cell viability was determined by two-way ANOVA followed by post-hoc Tukey’s test. Analysis was carried out using GraphPad Prism version 10.0.2 and a P value of <0.05 was considered statistically significant.

For analysis of proteomics data, both raw and normalized intensity protein ID and quantification tables were exported from Spectronaut for further analysis. Exploratory and technical analysis of data were carried out using in-house developed python pipeline with various visualisations using both the Plotly (RRID:SCR_013991) (24) and Pandas (RRID:SCR_018214) plotting libraries. Filtered data were exported from Perseus (25) into the R environment (v.4.31) for follow-up analyses. Protein abundance estimates were log2 transformed. PCA was calculated by replacing missing values with zeros and scaling the data. To evaluate the differences between samples at every time point and condition, pairwise T-tests were applied with the package rstatix (v.0.7.2). Benjamini-Hochberg correction for multiple comparisons was applied, with a false discovery rate (FDR) threshold of 0.05 for significance. Enrichment was calculated by first selecting proteins that were significantly different between conditions (FDR < 0.05) followed by the enrichment analysis in String DB (26) application programming interface (API) (v.11.5) with an in-house Python script, using Mus Musculus (NCBI Tax ID: 10090) as the database. Volcano and enrichment graphs were plotted with ggplot (v.3.4.4). Protein heat maps were created by calculating the ratio between treatment *vs*. control and representing them with ComplexHeatmap in R (2.16.0).

## Results

### A reduced protein diet halts tumor growth and induces ROS-mediated DNA damage

**Figure 1:**
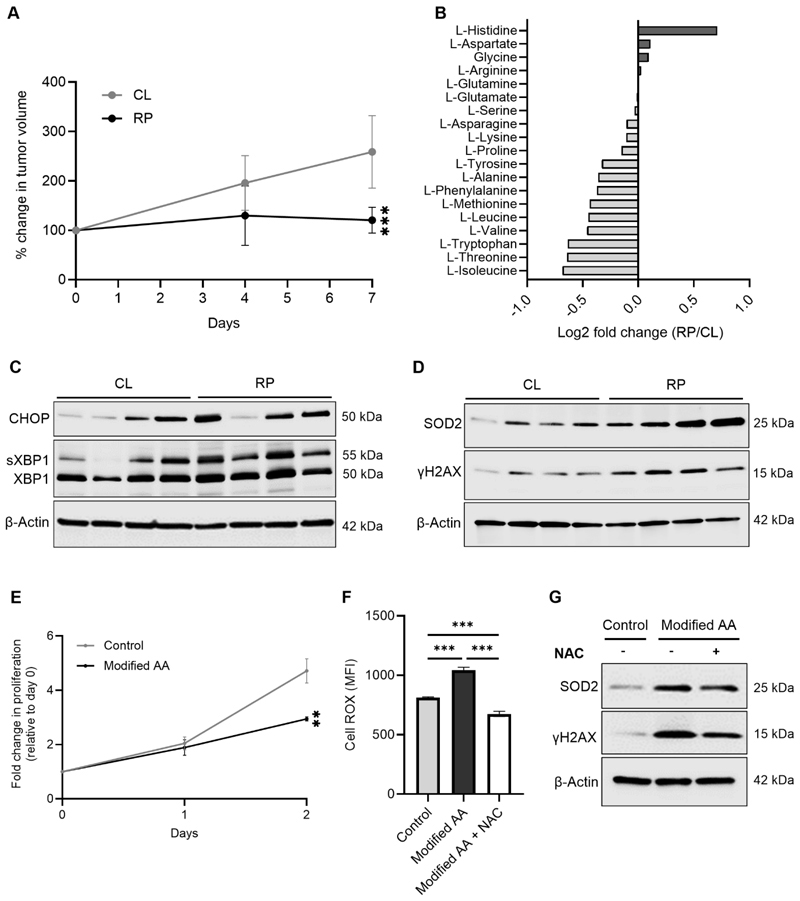
Reducing dietary protein suppresses growth of established tumors and induces DNA damage. **A**, Balb/c mice bearing CT26 subcutaneous tumors were fed control (CL) or 50% reduced protein diet (RP) and tumor volume was measured using calipers and the percent change in volume was calculated relative to day 0. n = 6 mice per group. **B**, Amino acid levels were measured in tumors from mice described in panel A on day 7 and reported as log2 fold change in the RP group compared to the CL group. n = 6 per group. **C-D**, Tumor-bearing mice were fed the same diets as in panel A, then western blots were performed to measure expression of protein markers of the endoplasmic reticulum (ER) stress response (**C**) and ER/mitochondrial-specific ROS and DNA damage (**D**) in tumors. **E**, CT26 cells were cultured in control or Modified amino acid (AA) media and the number of cells was quantified daily and reported as fold change compared to day 0 in each group. **F-G**, CT26 cells were cultured in control or Modified AA media with vehicle or Modified AA medium supplemented with 1 mM of N-acetylcysteine (NAC). ROS levels were determined 12 hrs later by flow cytometry using CellROX (**F**) and western blotting was performed on the indicated proteins 24 hrs later (**G**). Data are reported as mean ± S.D. **P<0.01; ***P<0.001.

To determine the impact of reducing dietary protein on the growth kinetics of established tumors, CT26 cells were inoculated into BALB/c mice and allowed to grow to a palpable size, while mice were fed control diet (CL). Mice were then randomized to either continue on CL diet or switched to an isocaloric diet in which protein content was reduced by 50% (RP diet) (Table S1). As shown in Fig. 1A, while tumors from mice continued on (CL grew by ~150% over 7 days, tumors from mice fed the 50% RP diet essentially stopped growing during this period. Intratumoral profiling of AA levels showed that the majority of AAs were decreased in abundance upon RP diet feeding (although histidine levels increased), compared to the CL group (Fig. 1B). Because nutrient restriction, including reduced availability of AAs, induces stress in the ER which triggers the UPR (27,28), protein expression of the UPR markers CHOP and sXBP1 were measured in tumors. Here, higher expression of each of these proteins was found in tumors from mice consuming the RP diet (Fig. 1C and Fig. S4A). In parallel with induction of the UPR, the protein levels of superoxide dismutase 2 (SOD2) and γH2AX, markers of ER/mitochondrial-specific ROS and DNA damage, respectively, were elevated (Fig. 1D and Fig. S4B).

In order to conduct mechanistic investigations into these pathways, an *in vitro* system was established in which CT26 cells were cultured in either control medium mimicking the levels of major metabolites (including AAs) found in HPLM (29) or the same medium in which AA levels were modified in accordance with the fold changes observed in tumors from RP diet-fed mice (Modified AA) (Fig. 1B; Table S2). Measurements of cell proliferation under these conditions revealed that modifying AA levels suppressed the proliferative rate of cells by nearly half compared to control conditions (Fig. 1E). Similar to what was observed *in vivo*, CT26 cells maintained in Modified AA medium increased ROS production and SOD2 and γH2AX expression, effects that were attenuated upon treatment with NAC (Fig. 1F and G).

### Reducing protein intake enhances the therapeutic efficacy of 5-FU in parallel with altering the tumor proteome

**Figure 2:**
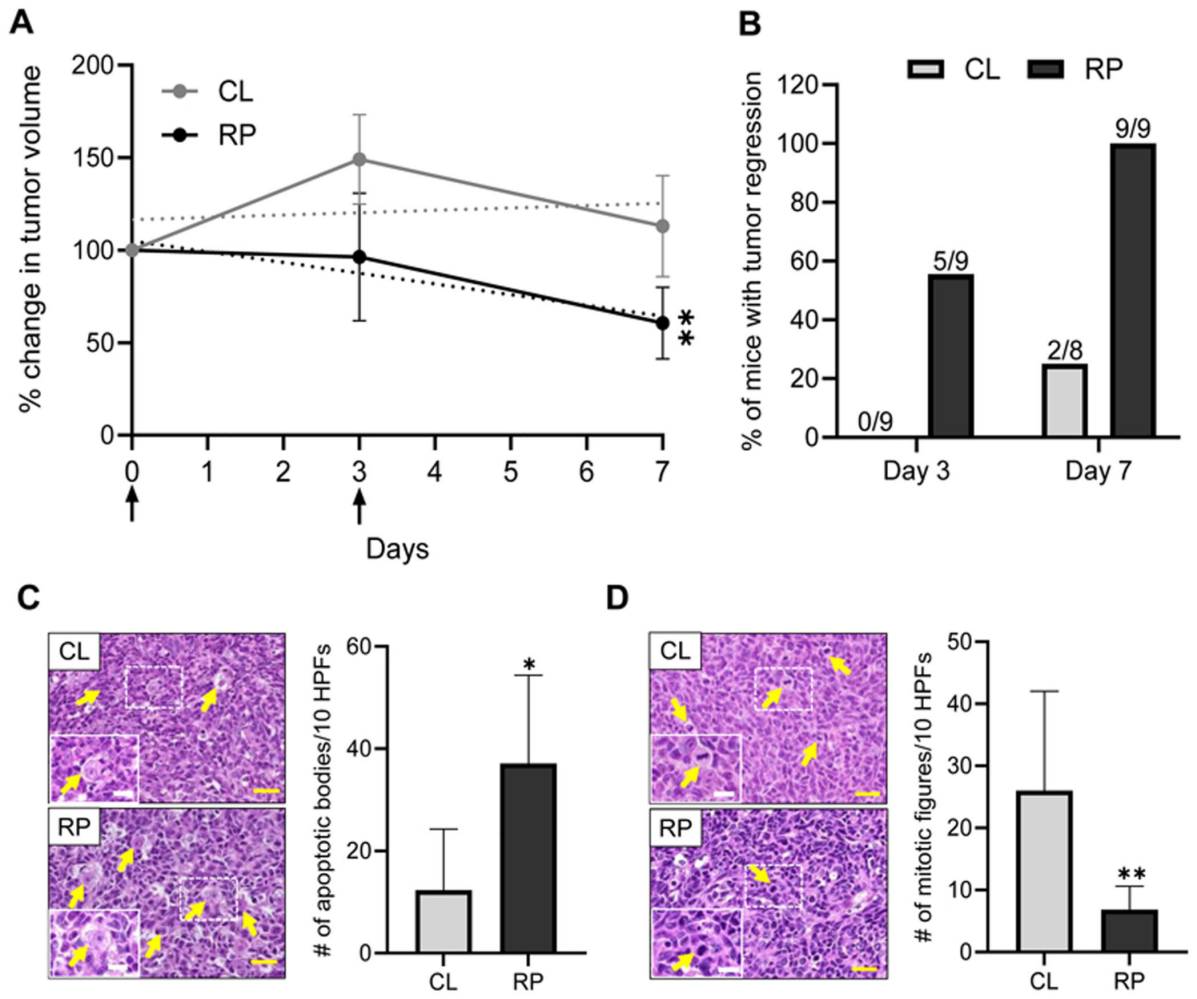
Feeding a reduced protein diet enhances tumor regression upon 5-FU treatment. **A**, Balb/c mice bearing CT26 subcutaneous tumors were fed control (CL) or 50% reduced protein diet (RP) and given two administrations of 5-FU (75mg/kg) (arrows). Tumor volume was measured using calipers and the percent change in tumor volume was calculated relative to day 0. Dashed lines indicate linear regression of average change in tumor volume for each group. N = 8-9 mice per group. **P<0.01 comparing regression lines over time. **B**, The percent of mice described in panel A that exhibited 10% or greater decrease in tumor volume was calculated on days 3 and 7. Values shown above bars indicate the number of mice with tumor regression over the total number of mice in the group. **C-D**, H&E-stained sections of tumors collected from mice described in Panel A were analyzed to determine the number of apoptotic bodies per 10 high power fields (HPFs) (**C**) and number of mitotic figures per 10 HPFs (**D**). Representative H&E-stained sections are shown on the left side and quantifications are shown on the right side, for each panel. Yellow arrows indicate apoptotic bodies and mitotic figures in panels C and D, respectively. Insets represent magnified view of the regions of interest indicated by dashed boxes. Yellow scale bar = 25 μm; white scale bar = 12.5 μm. n = 5-7 samples per group. Data are reported as mean ± S.D. *P≤0.05; **P<0.01.

Because of the observed increase in ER/mitochondrial stress, ROS and DNA damage in tumors following RP diet feeding, we reasoned that these changes would sensitize tumors to the chemotherapeutic agent 5-FU, a drug commonly used as frontline therapy to treat colorectal cancer (30). To test this, Balb/c mice bearing CT26 tumors were administered 5-FU upon initiating either diet. Measurements of changes in tumor size during a 7-day period showed that mice given a RP diet exhibited greater 5-FU induced regression compared to the CL group (Fig. 2A). Moreover, 55% of mice consuming the RP diet had 10% or greater tumor regression 3 days after the first dose, while all mice in that group had regression following two 5-FU doses (Fig. 2B). In contrast, no tumors from mice fed the CL diet regressed on day 3 and only 25% exhibited regression after two 5-FU treatments (Fig. 2B). Histologic analysis of tumors 7 days following initiation of 5-FU injections showed nearly 3 times as much apoptosis and more than a 3-fold reduction in mitosis (Fig. 2C and D). Importantly, although 5-FU treatments reduced the muscle weight of mice, this effect was not different between diet groups, suggesting that reducing protein intake does not enhance chemotherapy-induced cachexia (Fig. S5). A similar chemotherapeutic-enhancing effect of RP diet was observed upon Dox treatment in the murine orthotopic 4T1-derived breast cancer model, without adverse effects on muscle mass (Fig. S6A and B).

**Figure 3:**
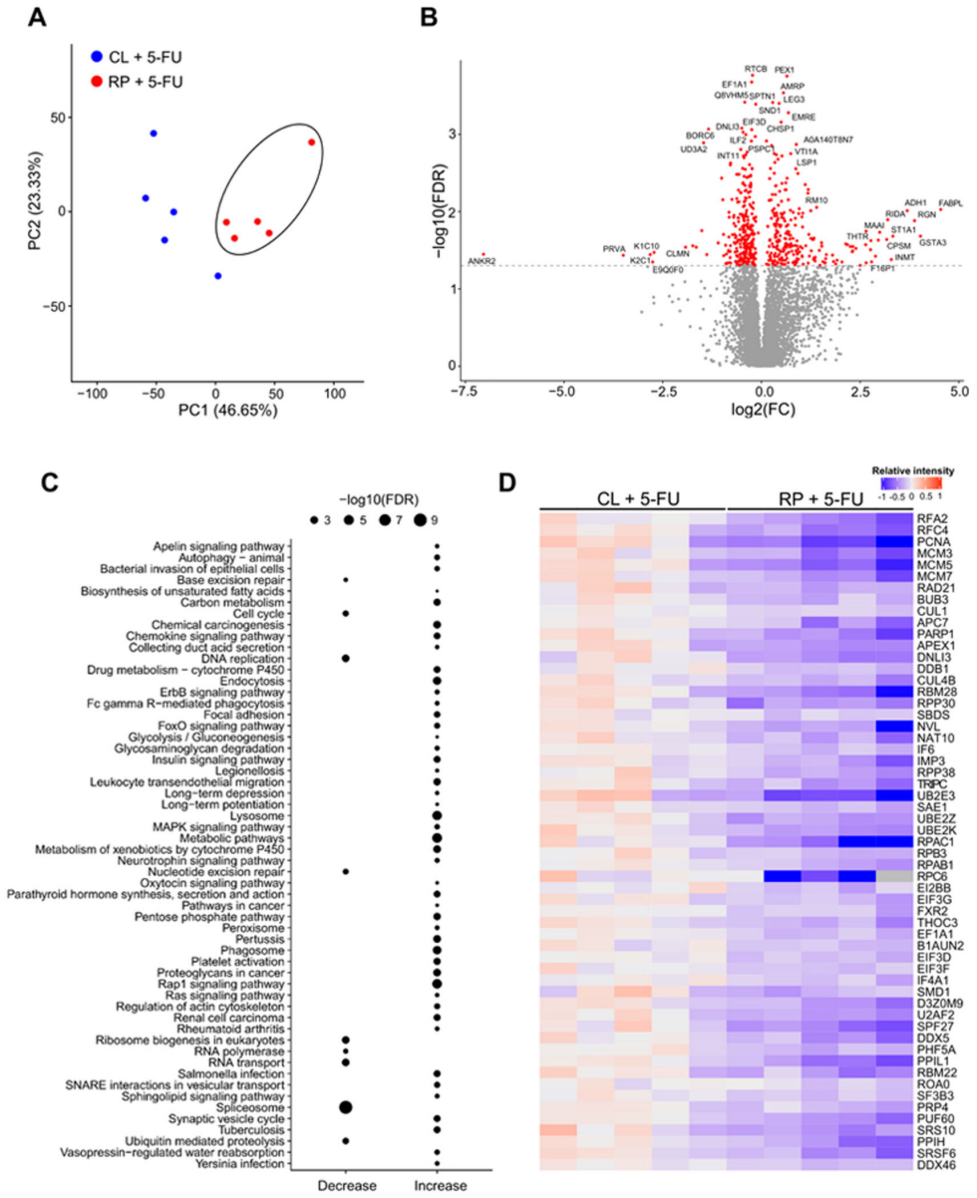
5-FU treatment exerts distinct effects on the tumor proteome in the presence of a reduced protein diet. Balb/c mice bearing CT26 subcutaneous tumors were fed control (CL) or reduced protein (RP) diets and administered 5-FU (75 mg/kg). Tumors were harvested for proteomic analysis 24 hours after 5-FU treatment. **A**, Principal component analysis of the proteomic data is shown. Black circle indicates distinct clustering of samples from the RP-fed group. **B**, Volcano plot displaying significantly increased or decreased abundance (red color) of proteins in tumors from mice fed a RP diet compared to mice fed a CL diet, is shown. **C**, Pathway analysis was conducted on the significantly changed proteins in tumors from mice fed a RP diet compared to mice fed CL diet, and shown as direction of change for each category. **D**, The relative levels of proteins within the pathways decreased in the RP diet group (as shown in Panel C), are displayed as a heatmap. Red color indicates higher levels and blue color indicates lower levels. Each column indicates an individual tumor.

To gain insight into the early molecular changes in tumors from 5-FU treated mice fed CL or RP diets, proteomics was conducted 24 hours following drug administration. Principal component analysis (PCA) revealed that samples from RP diet-fed mice clustered away from the CL diet group on principal component 1 (Fig. 3A). Examination of proteins that were significantly altered in abundance in the RP diet group showed 246 that increased and 264 that decreased, compared to the CL group (Fig. 3B) (ProteomeXchange, PXD052110). Subsequent pathway analysis of these changes revealed several that were decreased, which were related to base and nucleotide excision repair, DNA replication and cell cycle, as well as protein and RNA production and transport (Fig. 3C and D). Numerous pathways were also increased in the RP group, which mainly included those related to metabolism (Fig. 3C and Fig. S7). Because DNA stress can trigger the mTORC1 pathway (31,32), we measured phosphorylated P70S6K protein expression, a key marker of mTORC1 activation (15), however no significant difference was observed upon dietary protein reduction and/or 5-FU treatment (Fig. S8)

### Reducing AA availability during 5-FU treatment maximally induces the cGAS/STING1 pathway

**Figure 4:**
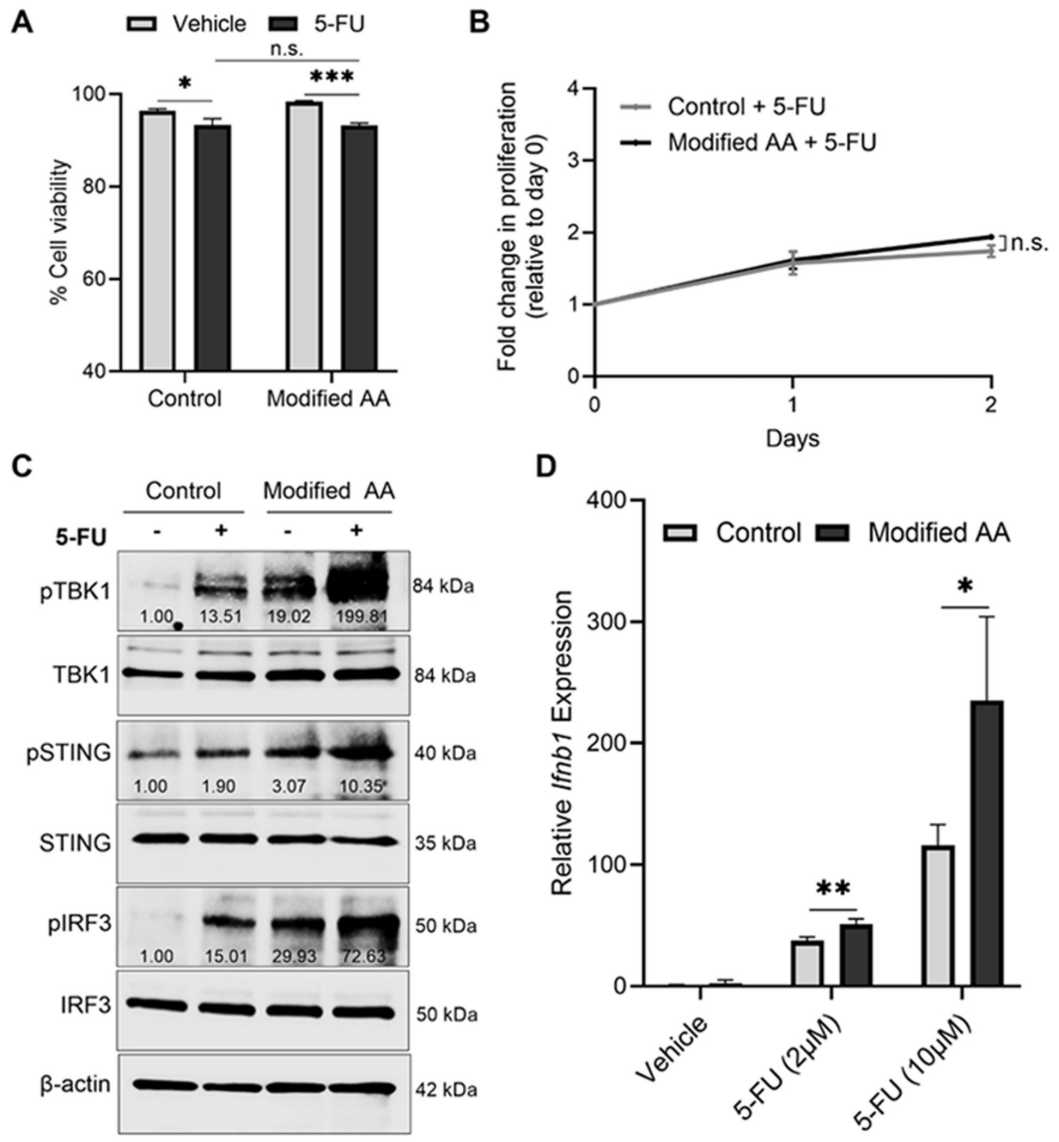
Reducing amino acid availability enhances the activation of the cGAS-STING pathway in colon cancer cells following 5-FU treatment. **A**, CT26 cells were cultured in control or Modified amino acid (AA) media and treated with either vehicle or 5-FU (10μM) for 72 h and viability was determined by flow cytometry following DiOC6 and DAPI staining. Data are reported as mean ± S.D. n = 3 samples per group. **B**, CT26 cells were cultured in control or Modified AA media and treated with 5-FU (10μM) and the number of cells were quantified daily and reported as fold change compared to day 0 in each group. n.s. = not significant comparing regression lines over time. **C**, CT26 cells were cultured in control or Modified AA media and treated with either vehicle or 5-FU for 6 h and western blotting was performed on the indicated proteins. Values shown under bands represent fold change of phospho protein expression compared to the respective total protein, normalized to β-Actin. **D**, CT26 cells were cultured in control or Modified AA media and treated with either vehicle or 5-FU for 6 h then all groups were switched to complete RPMI medium for an additional 18 h. qRT-PCR was then performed to determine the relative expression of *Ifnb1*. Data are reported as mean ± S.D. n = 3 samples per group. Data are reported as mean ± S.D. *P≤0.05; **P<0.01; ***P<0.001; ****P<0.0001. n.s. = not significant.

Given the enhanced anti-tumor efficacy of 5-FU and proteome alterations in the context of a RP diet, we next evaluated whether limiting AA availability directly rendered tumor cells more susceptible to the cytotoxic effects of chemotherapy. To investigate this, the *in vitro* system described in Fig. 1 was employed in order to eliminate potential contributions from the tumor microenvironment or systemic factors. Here, we found that although 5-FU caused a significant decrease in the viability of CT26 cells, this effect was not enhanced when cells were cultured in modified AA medium (Fig. 4A). Further, no difference in the proliferation of cells was observed upon 5-FU treatment in either culture condition (Fig. 4B). Because these findings suggest that 5-FU may not be exerting greater anti-tumor effects under reduced AA availability through tumor cell-intrinsic mechanisms, we next investigated whether tumor cell-mediated modulation of the microenvironment may be a contributing factor. Evidence suggests that reducing dietary protein enhances the anti-tumor immune response (17,18). Further, 5-FU can mediate aspects of its anti-cancer effects through the immune-activating cGAS/STING1 pathway (33). To test whether cGAS/STING1 is activated under AA-modified conditions in the presence or absence of 5-FU, CT26 cells were maintained under control or modified AA conditions and treated with vehicle or 5-FU. Measurements of the phosphorylated forms of TBK1, STING1 and IRF3 revealed increased expression in cells cultured in the Modified AA medium, compared to control medium, when both groups were given vehicle (Fig. 4C). Although 5-FU treatment increased the expression of these proteins compared to vehicle treatment in cells from either medium, this elevation was augmented in the Modified AA group (Fig. 4C). A similar enhancement was observed for the transcription *Ifnb1*, the immune-stimulating Type I interferon (IFN) downstream of cGAS/STING1 activation (34) (Fig. 4D).

### Reducing dietary protein enhances 5-FU mediated anti-tumor immunity

**Figure 5:**
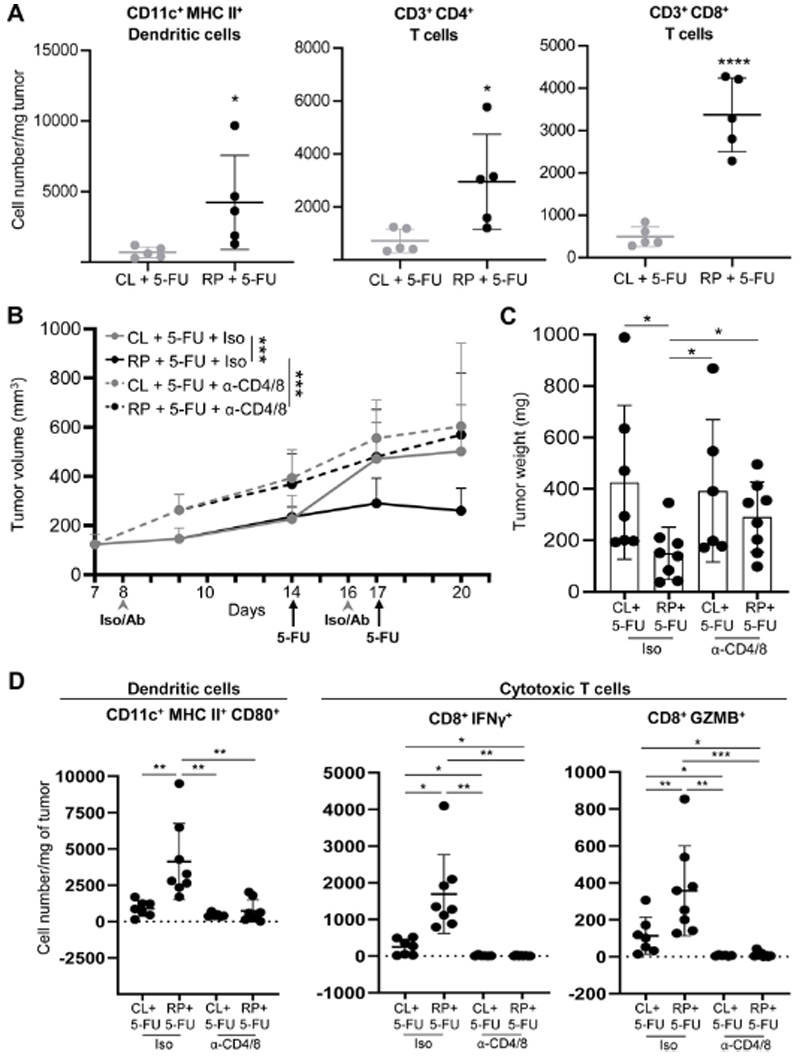
Combining dietary protein reduction with 5-FU enhances immune-dependent tumor growth suppression. **A**, Balb/c mice bearing CT26 subcutaneous tumors were fed control (CL) or 50% reduced protein (RP) diet for 5 days while receiving 2 injections of 5-FU (75 mg/kg). Tumors were harvested on day 5 to quantify the abundance of dendritic cells (left), CD4^+^ T cells (middle) and CD8^+^ T cells (right) per mg of tumor. n = 5 per group. **B**, CT26 were injected subcutaneously in Balb/c mice and allowed to grow for 14 days, and mice were randomized to receive either isotype control (IgG) (Iso) or CD4 and CD8 blocking antibodies (Ab), as indicated. Mice in each group were further stratified to receive either CL or RP diets for 5 days and 2 injections of 5-FU (75 mg/kg), as indicated. Tumor volume was measured over a 20 day period. n= 7-8 mice per group. **C**, Tumor weights were measured in tumors from mice described in panel B on day 20. **D**, The abundance of activated dendritic cells, and activated cytotoxic T cells were quantified by flow cytometry in tumors from mice described in panel B, on day 20. N=7-8 mice per group. Data are reported as mean ± S.D. *P≤0.05; **P<0.01; ***P<0.001; ****P<0.0001.

To determine whether the observed induction of the cGAS/STING1 pathway under AA-modified conditions correlates with greater anti-tumor immunity, mice bearing CT26 tumors were fed CL or RP diets and treated with 5-FU. Flow cytometric analysis of intratumoral immune cell populations revealed significantly higher numbers of dendritic cells (DCs) and helper CD4^+^ and cytotoxic CD8^+^ T cells (Fig. 5A). Given this observation, we next sought to determine whether enhanced immune cell infiltration was causally linked to tumor suppression. To evaluate this, tumor-bearing Balb/c mice were fed CL or RP diets and treated with 5-FU while receiving either control IgG or CD4 and CD8 depleting antibodies. This revealed that while the RP diet again suppressed tumor growth compared to the CL diet, depleting CD4^+^ and CD8^+^ T cells reversed that effect (Fig 5B and C). Further analysis of immune cell populations in tumors from these groups showed significantly higher abundance of CD80+ DCs, and interferon gamma (IFNγ) and granzyme B (GZMB)-positive CD8^+^ T cells in the RP group given isotype compared to the CL group given isotype, while these populations were nearly undetectable in either diet group given CD4/CD8 depleting antibodies(Fig 5D). Reduced populations of CD4^+^ and CD8^+^ T cells following administration of depleting antibodies was also confirmed in spleens (Fig. S9). Notably, no difference in the length of the muscle cross-section was observed in mice fed either diet and treated with 5-FU and isotype control (Fig. S10).

## Discussion

Although nutrient deprivation is a promising complementary anti-cancer approach, the optimal diet-based method to accomplish this goal is not well understood. Here, we demonstrate that reducing dietary protein by 50% in mice induces a stress response with accompanying ROS-mediated DNA damage. Further, this diet sensitizes tumors to 5-FU induced regression, and proteome alterations. Notably, the 5-FU mediated effects on tumor cells were not exerted directly, but instead activated the cGAS/STING1 pathway and promoted anti-tumor immunity. Taken together, these findings suggest that reducing protein intake could improve therapeutic response in patients with cancer.

Previous work conducted in immunodeficient mice demonstrated that the growth of tumors in models of breast and prostate cancer was suppressed when dietary protein was reduced by nearly 70% (15,16). Interestingly, this tumor-suppressive effect occurred irrespective of whether diet was administered simultaneously with tumor cell inoculation or after tumors were already established, although the low protein diet was more effective in the former condition (15,16). Notably, reducing dietary protein in this manner decreased circulating IGF-1 levels and mTORC1 activity (15,16). Our work found that mTORC1 activity, as determined by expression of phosphorylated P70S6K, was not significantly changed in tumors when the RP diet was given in the presence or absence of 5-FU despite observing a strong reduction in the levels of intratumoral AAs. This lack of effect may have resulted from either a more modest reduction in dietary protein compared to previous work, and/or the simultaneous activation of mTORC1 in response to the DNA damage occurring in this setting (15,31,32). This DNA damage likely results from increased ER/mitochondrial-specific ROS production (indicated by elevated SOD2 levels). However, it should be noted that although DNA damage correlated with this increase, NAC treatment only partially attenuated the enhanced expression of γH2AX upon AA modulation *in vitro*, suggesting that DNA damage occurs in this context through additional mechanisms, possibly from reduced nucleotide pools (35,36). Regardless, it is likely that increased DNA damage during AA restriction is likely playing a role in suppressing tumor growth. In light of this, we reasoned that this level of stress would render tumor cells more sensitive to chemotherapy. Interestingly, despite these observed abnormalities, direct administration of 5-FU to cells in Modified AA medium did not enhance cell death or further suppress proliferation. Although these findings support a role for tumor cell-extrinsic mediators in enhancing the anti-tumors effects of 5-FU, it is also possible that long-term culture of cells under these conditions could result in cancer cell death given that prolonged activation of the UPR can promote apoptosis (37,38). Moreover, the observed decrease in pathways related to DNA repair and proliferation in tumors from mice fed RP and given 5-FU, as revealed by proteomics, could contribute to tumor-cell intrinsic defects over a longer period.

In addition to the ability of a reduced protein diet to decrease circulating growth factors and disrupt tumor cell intrinsic growth-promoting pathways, recent evidence suggests that this diet alteration can enhance anti-tumor immunity. For instance, tumor suppression was observed upon lowering protein intake in immunocompetent mice using syngeneic tumor models, an effect that was reversed upon depletion or neutralization of CD8_+_ T cells or antigen presenting cells (18). Consistent with these findings and a subsequent study by the same group, tumors from mice fed the low protein diet had higher infiltration of CD8_+_ T cells and myeloid cells (18,39). The work carried out in the current study found that when combined with 5-FU, the RP diet induced up to a 7-fold increase in CD4^+^ and CD8^+^ T cells as well as dendritic cells. To our knowledge, this is the first demonstration that reducing protein intake can enhance immune infiltration of tumors in the presence of chemotherapy. Additional data demonstrating that depleting CD4^+^ and CD8^+^ T cells reverses the antitumor effects of combining a RP diet with 5-FU, strongly suggest that the observed immune response is largely responsible for the enhanced anti-tumor effects of this combination. Moreover, previous work showing that feeding a diet with reduced levels of non-essential amino acids to immunodeficient mice did not enhance suppression of tumor growth during treatment with the chemotherapeutic agents gemcitabine or paclitaxel, further supports this concept (40). Notably, reducing protein intake in mice enhances the anti-tumor response to immunotherapy, further supporting the connection between reducing this macronutrient and improving immune-mediated tumor suppression (17). Although the exact mechanistic underpinnings of the immune-promoting effect of the RP diet during 5-FU treatment remains to be formally investigated, it is reasonable to posit that activation of the UPR, mediated by IRE1α, plays a role given previous findings (18,39) and the observed increase in CHOP and sXBP1 in tumors from RP-fed mice in the current work. However, a novel possibility is that activation of the cGAS/STING1 pathway is, at least, partially responsible. This concept is not only supported by the observed increase in activation of key components of this pathway, including *Ifnb1*, in cancer cells cultured under AA modified conditions, but recent evidence that 5-FU independently exerts its anti-tumor effects through cGAS/STING1 (33). Although further work is needed to directly determine the contribution of cGAS/STING1 to the diet-mediated enhancement of chemotherapeutic efficacy observed in the current study, it is likely that this pathway is relevant given the large body of literature supporting its role in recruiting tumor-killing immune cells (41).

Although ample evidence supports oncogenic roles of individual AAs, restricting single or multiple select AAs is not easily feasible in humans. Therefore, reducing whole protein, which contains these metabolites, may be a promising alternative. In fact, dietary intervention trials have been conducted to achieve this goal, albeit not in cancer patients, which have demonstrated physiologic effects including reduced circulating and gut luminal AA levels (42,43). One concern that may arise when considering reducing protein intake in cancer patients receiving chemotherapy is the possibility of inducing cachexia, given the ability of the disease and/or treatment to induce this condition, and the importance of dietary protein for maintaining muscle mass (44). However, in mice bearing CT26 or 4T1-derived tumors, reducing dietary protein by 50% did not exacerbate the 5-FU induced decrease in muscle mass. This finding is even more relevant given that the CT26 tumor model has a propensity to develop cachexia (45,46). Therefore this type of dietary intervention may be safe (at least in patients with non-advanced, non-metastatic disease) if protein intake is kept within the limits of recommended daily allowance. Another consideration for modifying the amount of any macronutrient as a dietary intervention is how other dietary components will be altered to ensure sufficient caloric intake. Many murine tumor studies elevate carbohydrate in the form of corn starch in protein-reduced diets to make them isocaloric with the respective control diet (15–17). The current work utilized a diet in which fat, in the form of soybean oil, was increased to make the RP diet isocaloric with the control diet. Soybean oil, the typical fat source in standard purified diets for mice, is a largely unsaturated ‘healthy’ fat unlikely to exert protumorigenic or other adverse effects. Further, we avoided increasing carbohydrates given the propensity of this macronutrient to promote tumorigenesis (47–50). We believe that a similar approach could be taken in humans by providing an unsaturated fat to account for calories lost from reduced protein intake, which was done in a recent controlled feeding trial in cancer-free individuals (42). Importantly, the work carried out here implemented the dietary modification and chemotherapeutic treatment in mice with already established tumors, thus mimicking a clinical scenario, further highlighting the relevance of the findings described here for cancer patients. Collectively, the cancer-focused work in mice shown in the current study and previous reports, along with the feasibility of reducing dietary protein in humans, provide a rationale for testing whether a reduced protein diet exerts anti-cancer effects in patients.

## Supplementary Material

Supplementary Information

## Data Availability

All raw data generated in this study are available upon request from the corresponding author. The proteomics data can be found at www.proteomexchange.org under the identifier: PXD052110.
