# Supplementary material for: Reducing Dietary Protein Enhances the Antitumor Effects of Chemotherapy through Immune-Mediated Mechanisms
Source: Mol Cancer Ther. Author manuscript; Available in PMC 2025 Apr 24. (PMC7617599; doi:10.1158/1535-7163.MCT-24-0545)
Supplement: Supplementary Information [file EMS204084-supplement-Supplementary_Information.zip › supp_info_12(1).docx]

| **Table S2. Composition of cell culture media** | | |
| --- | --- | --- |
| **Component** | **Control** | **Modified AA** |
|  | mM | mM |
| Alanine | 0.43 | 0.28 |
| Cysteine | 0.04 | 0.04 |
| Aspartic acid | 0.02 | 0.02 |
| Glutamic acid | 0.08 | 0.08 |
| Glycine | 0.3 | 0.33 |
| Asparagine | 0.05 | 0.04 |
| Proline | 0.2 | 0.17 |
| Glutamine | 0.55 | 0.55 |
| Arginine | 0.11 | 0.11 |
| Serine | 0.15 | 0.14 |
| Tyrosine | 0.08 | 0.05 |
| Cystine | 0.1 | 0.10 |
| Phenylalanine | 0.08 | 0.05 |
| Histidine | 0.11 | 0.19 |
| Isoleucine | 0.07 | 0.02 |
| Lysine | 0.2 | 0.18 |
| Leucine | 0.16 | 0.09 |
| Methionine | 0.03 | 0.02 |
| Threonine | 0.14 | 0.05 |
| Valine | 0.22 | 0.12 |
| Tryptophan | 0.06 | 0.02 |
| Glucose | 5 | |
| Sodium pyruvate | 0.05 | |
| HEPES | 15 | |
| Sodium bicarbonate | 23.81 | |
