# Supplementary material for: Reducing Dietary Protein Enhances the Antitumor Effects of Chemotherapy through Immune-Mediated Mechanisms
Source: Mol Cancer Ther. Author manuscript; Available in PMC 2025 Apr 24. (PMC7617599; doi:10.1158/1535-7163.MCT-24-0545)
Supplement: Supplementary Information [file EMS204084-supplement-Supplementary_Information.zip › supp_info_11.docx]

| **Table S1. Composition of experimental diets.** | | |
| --- | --- | --- |
|  | **Control (CL)** | **Reduced Protein (RP)** |
| **Ingredient** | **gm** | **gm** |
| Casein | 200 | 100 |
| L-Cystine | 3 | 1.5 |
| Corn Starch | 397.48 | 397.48 |
| Maltodextrin 10 | 132 | 132 |
| Sucrose | 107.07 | 107.07 |
| Cellulose, BW200 | 50 | 50 |
| Soybean Oil | 70 | 109.3 |
| t-Butylhydroquinone | 0.014 | 0.014 |
| Mineral Mix S10022G | 3.5 | 3.5 |
| Calcium Phosphate, Dibasic | 0 | 3 |
| Calcium Carbonate | 12.495 | 9.5 |
| Potassium Citrate, Monohydrate | 2.4773 | 2.3 |
| Potassium Phosphate, Monobasic | 6.86 | 6.5 |
| Sodium Chloride | 2.59 | 2.59 |
| Vitamin Mix V10037 | 10 | 10 |
| Choline Bitartrate | 2.5 | 2.5 |
| **Total** | **1000** | **937.26** |
| Protein (gm) | 177.0 | 88.5 |
| Carbohydrate (gm) | 646.6 | 646.6 |
| Fat (gm) | 70.0 | 109.3 |
| Fiber (gm) | 50.0 | 50.0 |
| Protein (kcal) | 708 | 354 |
| Carbohydrate (kcal) | 2586 | 2586 |
| Fat (kcal) | 630 | 984 |
| **Total** | **3924** | **3924** |
| Protein (gm%) | 18 | 9 |
| Carbohydrate (gm%) | 65 | 69 |
| Fat (gm%) | 7 | 12 |
| Protein (kcal%) | 18 | 9 |
| Carbohydrate (kcal%) | 66 | 66 |
| Fat (kcal%) | 16 | 25 |
| Calcium (g/kg) | 5.0 | 5.0 |
| Phosphorus (g/kg) | 3.2 | 3.2 |
| Potassium (g/kg) | 3.6 | 3.7 |
| **kcal/gm** | **3.9** | **4.2** |
