# Supplementary figures and images for: Reducing Dietary Protein Enhances the Antitumor Effects of Chemotherapy through Immune-Mediated Mechanisms
Source: Mol Cancer Ther. Author manuscript; Available in PMC 2025 Apr 24. (PMC7617599; doi:10.1158/1535-7163.MCT-24-0545)

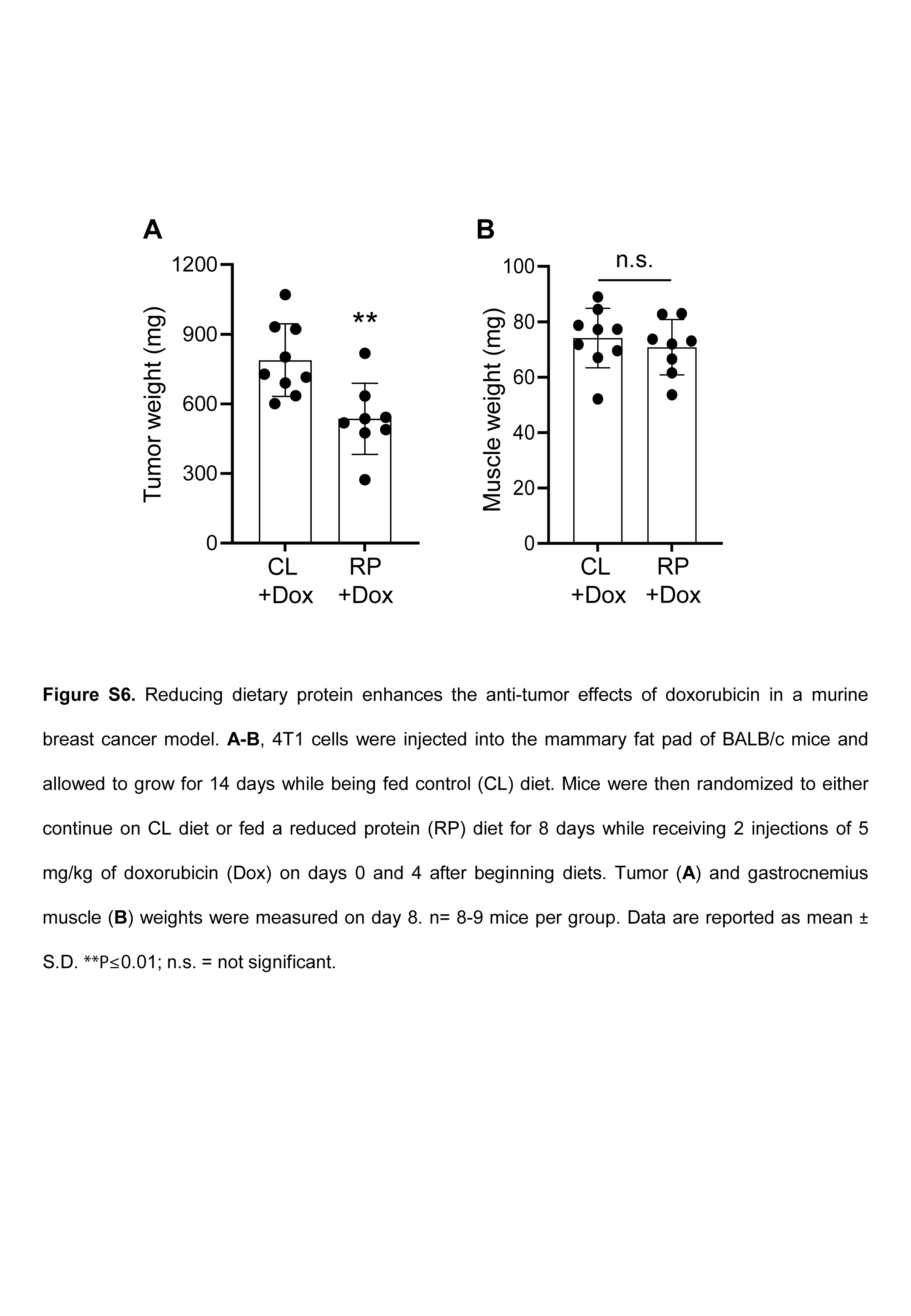

Supplement: Supplementary Information [file EMS204084-supplement-Supplementary_Information.zip › supp_info_6.tif]

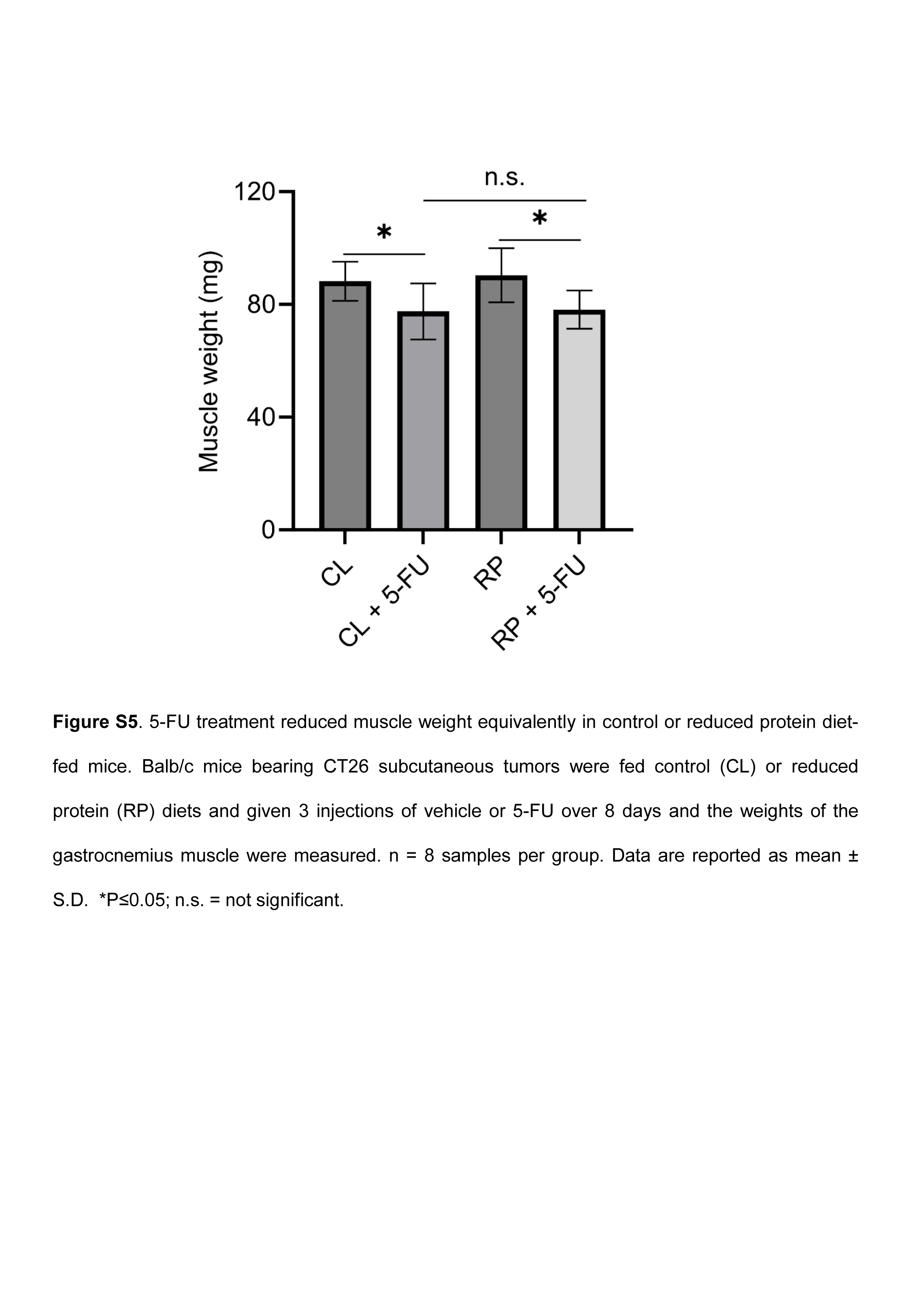

Supplement: Supplementary Information [file EMS204084-supplement-Supplementary_Information.zip › supp_info_5.tif]

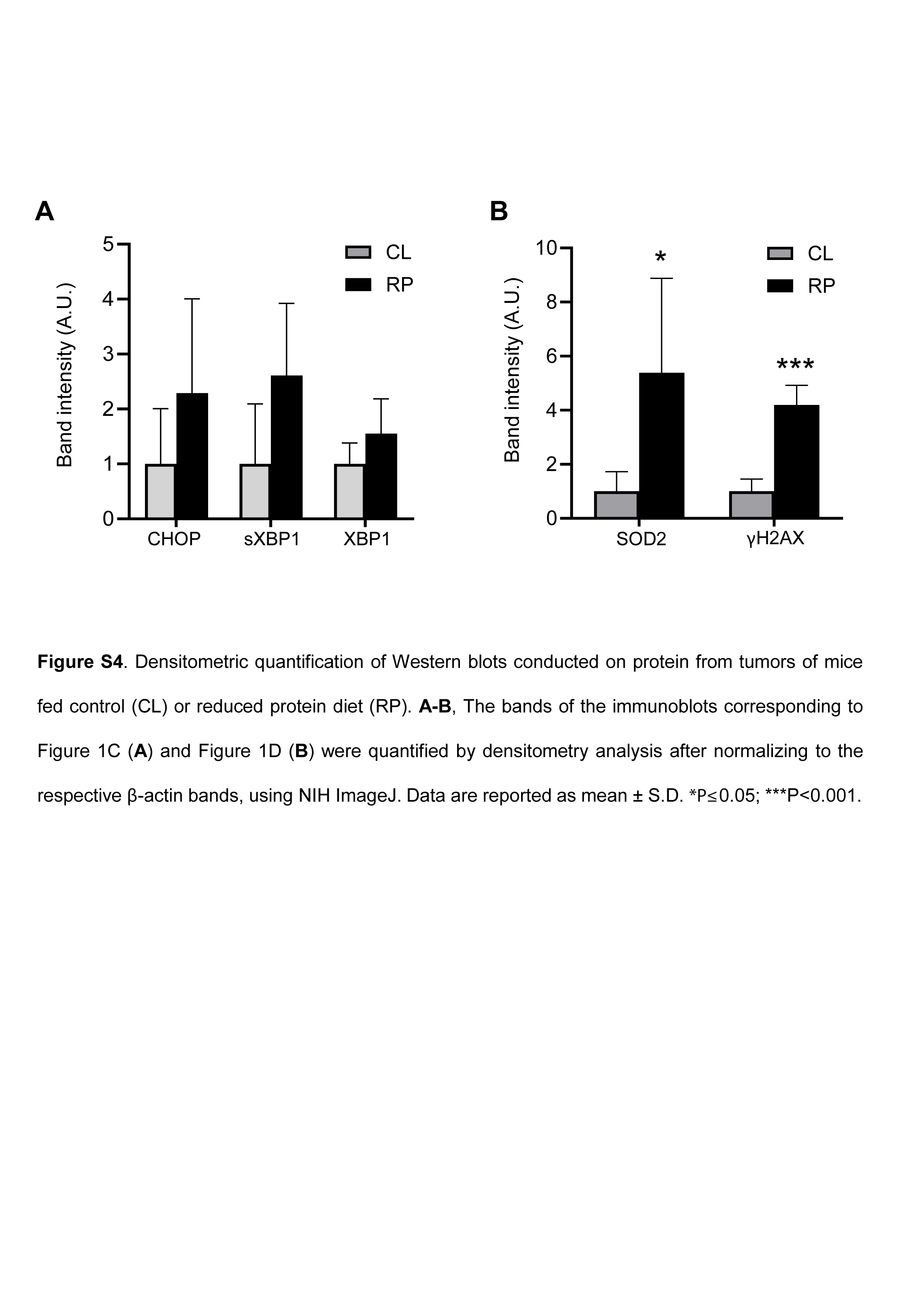

Supplement: Supplementary Information [file EMS204084-supplement-Supplementary_Information.zip › supp_info_4.tif]

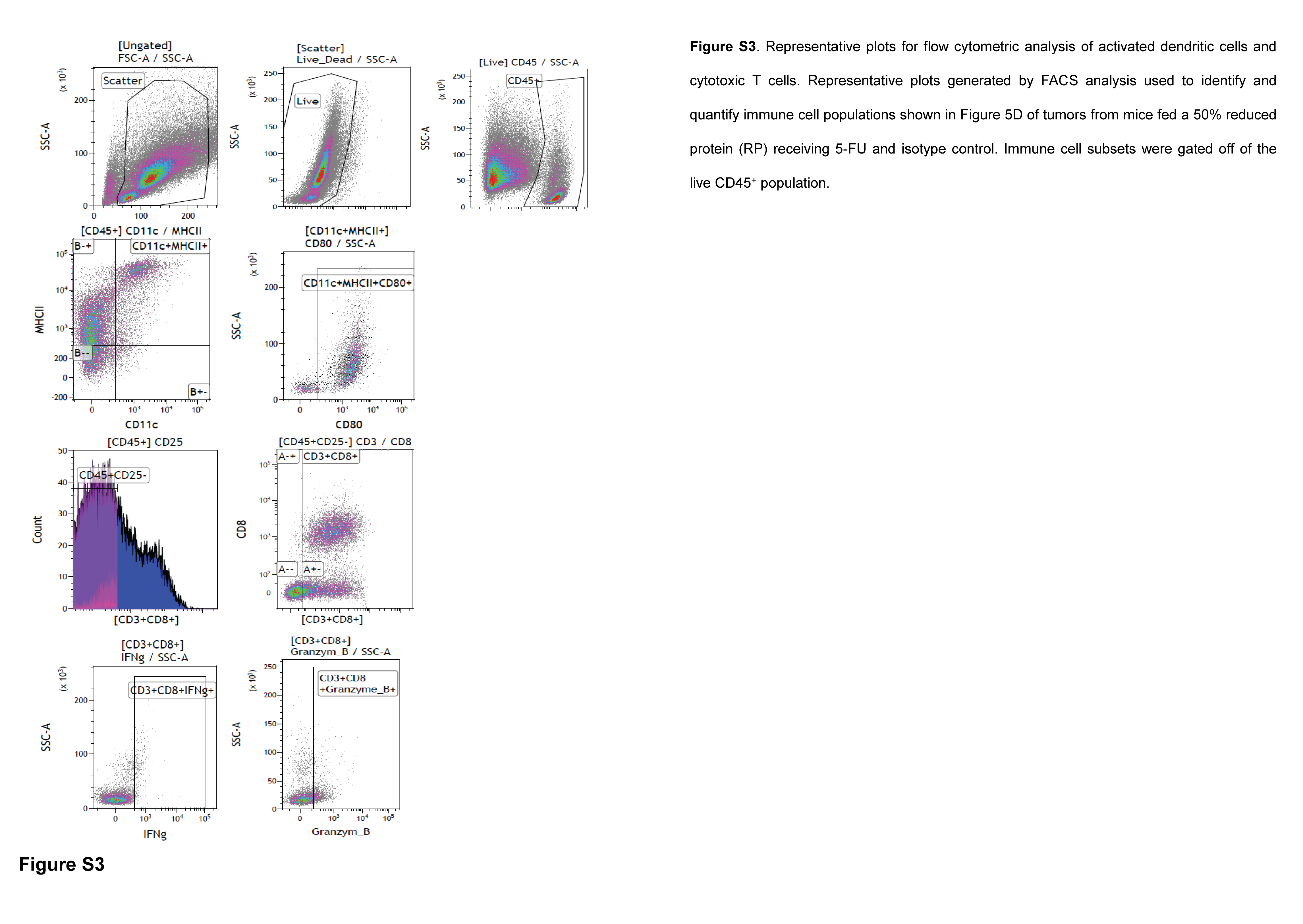

Supplement: Supplementary Information [file EMS204084-supplement-Supplementary_Information.zip › supp_info_3.tif]

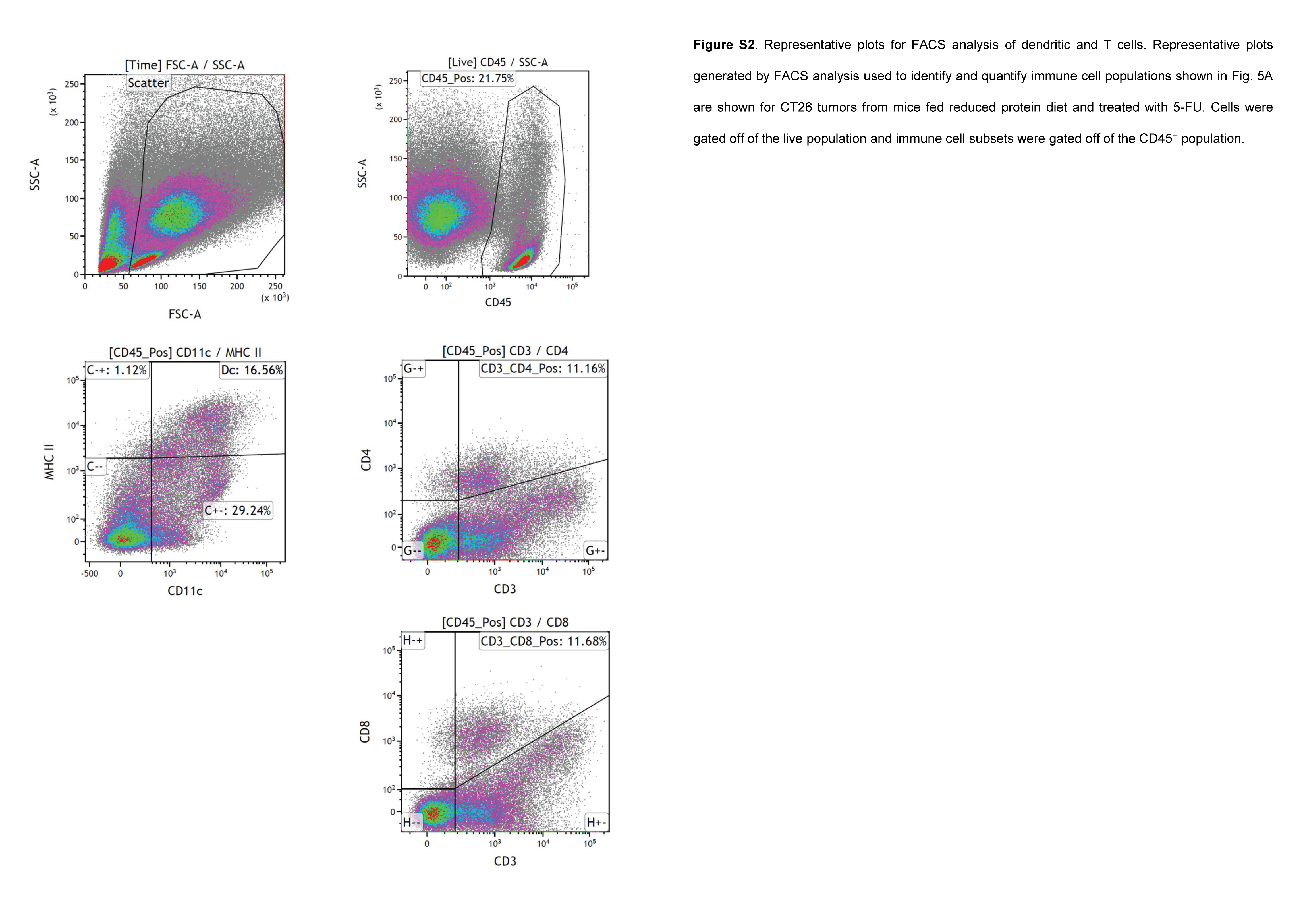

Supplement: Supplementary Information [file EMS204084-supplement-Supplementary_Information.zip › supp_info_2.tif]

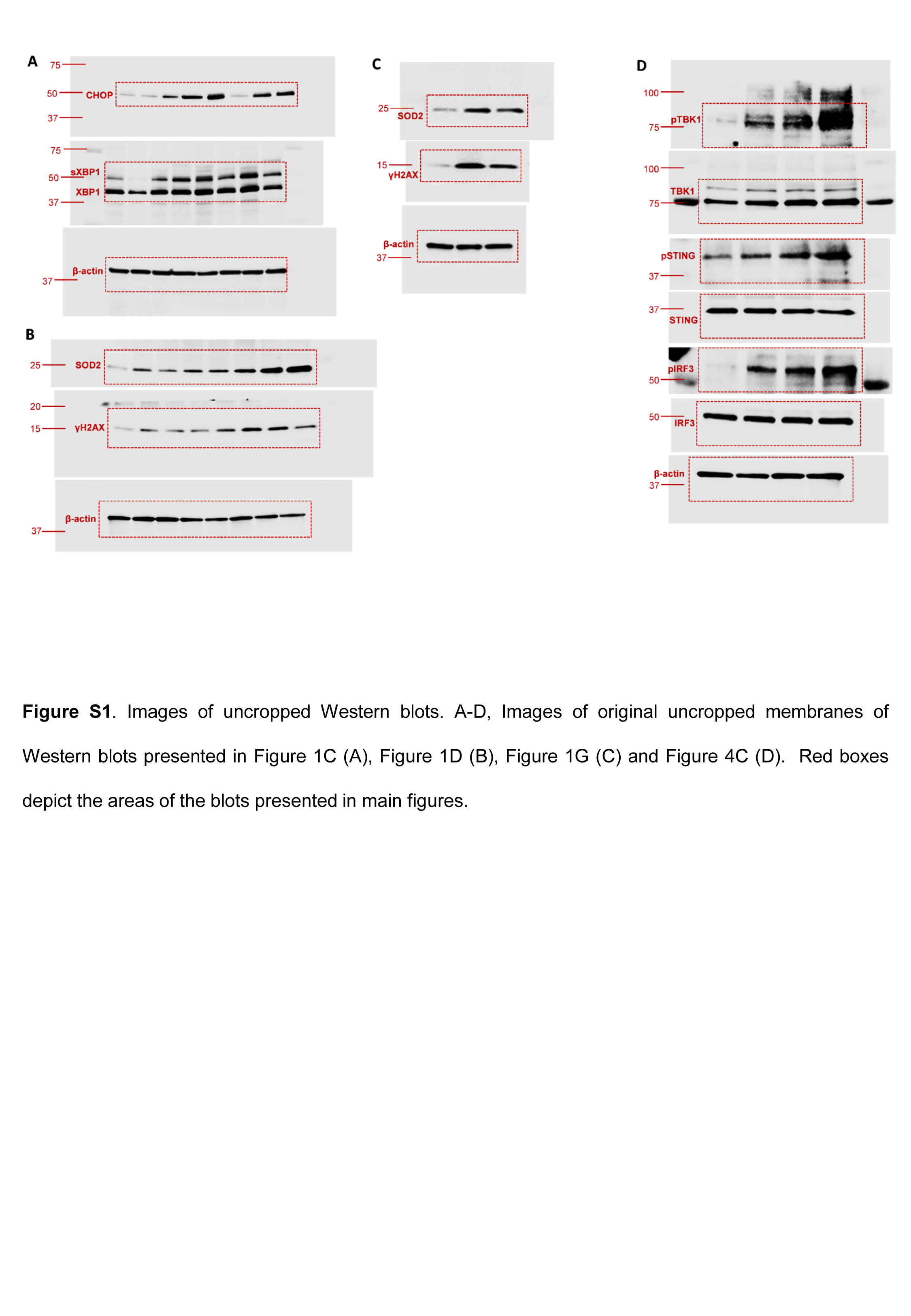

Supplement: Supplementary Information [file EMS204084-supplement-Supplementary_Information.zip › supp_info_1.tif]

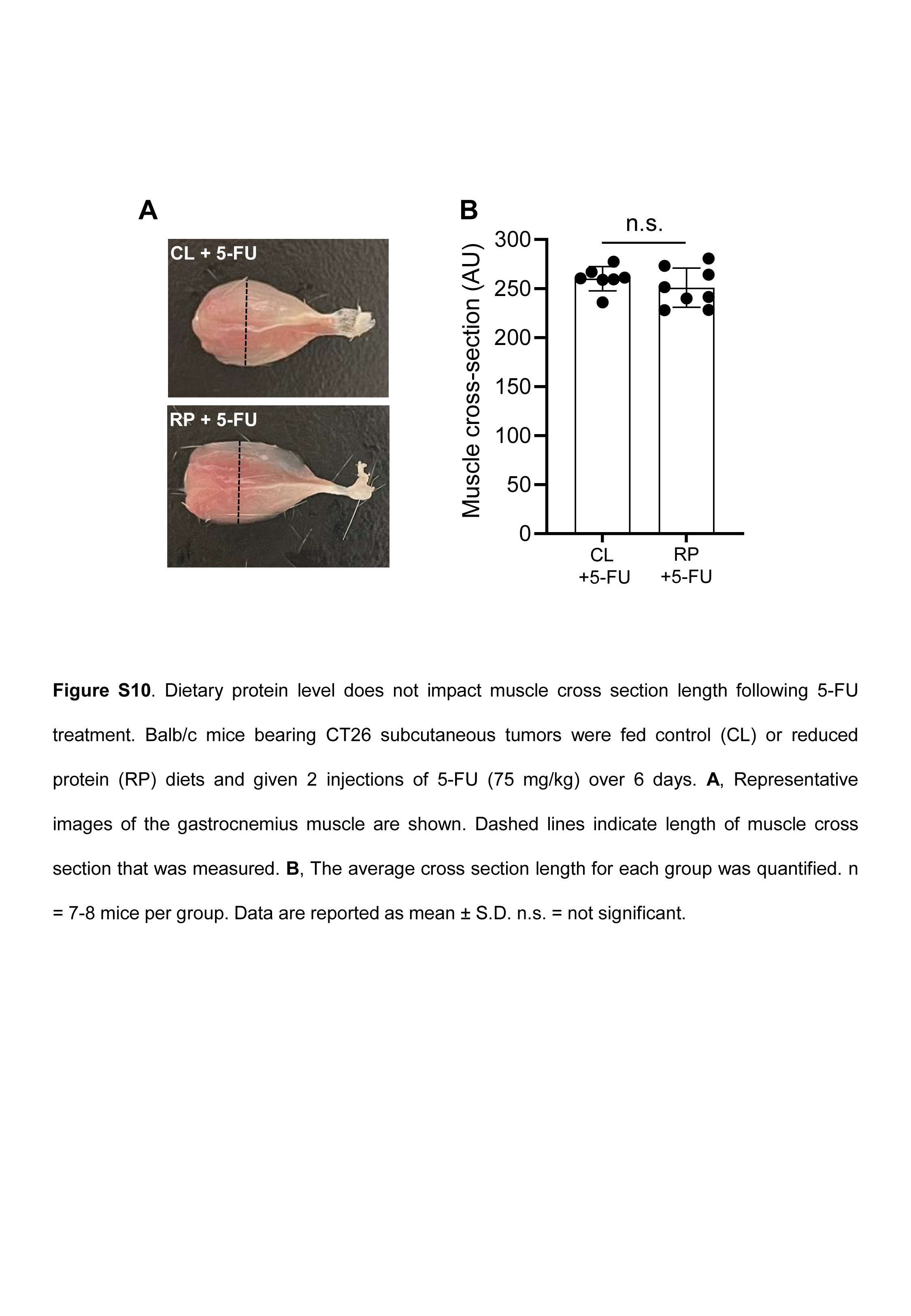

Supplement: Supplementary Information [file EMS204084-supplement-Supplementary_Information.zip › supp_info_10.tif]

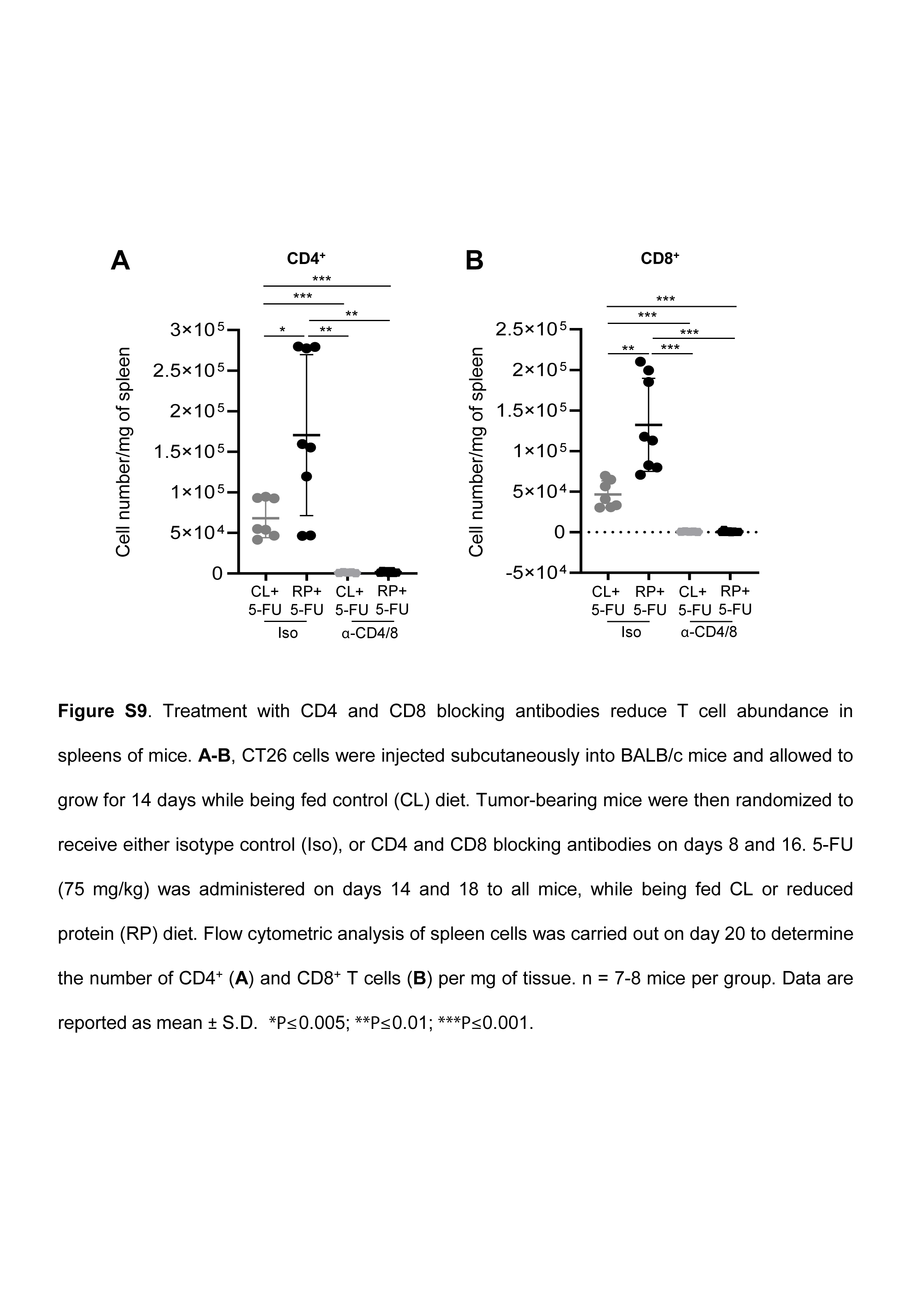

Supplement: Supplementary Information [file EMS204084-supplement-Supplementary_Information.zip › supp_info_9.tif]

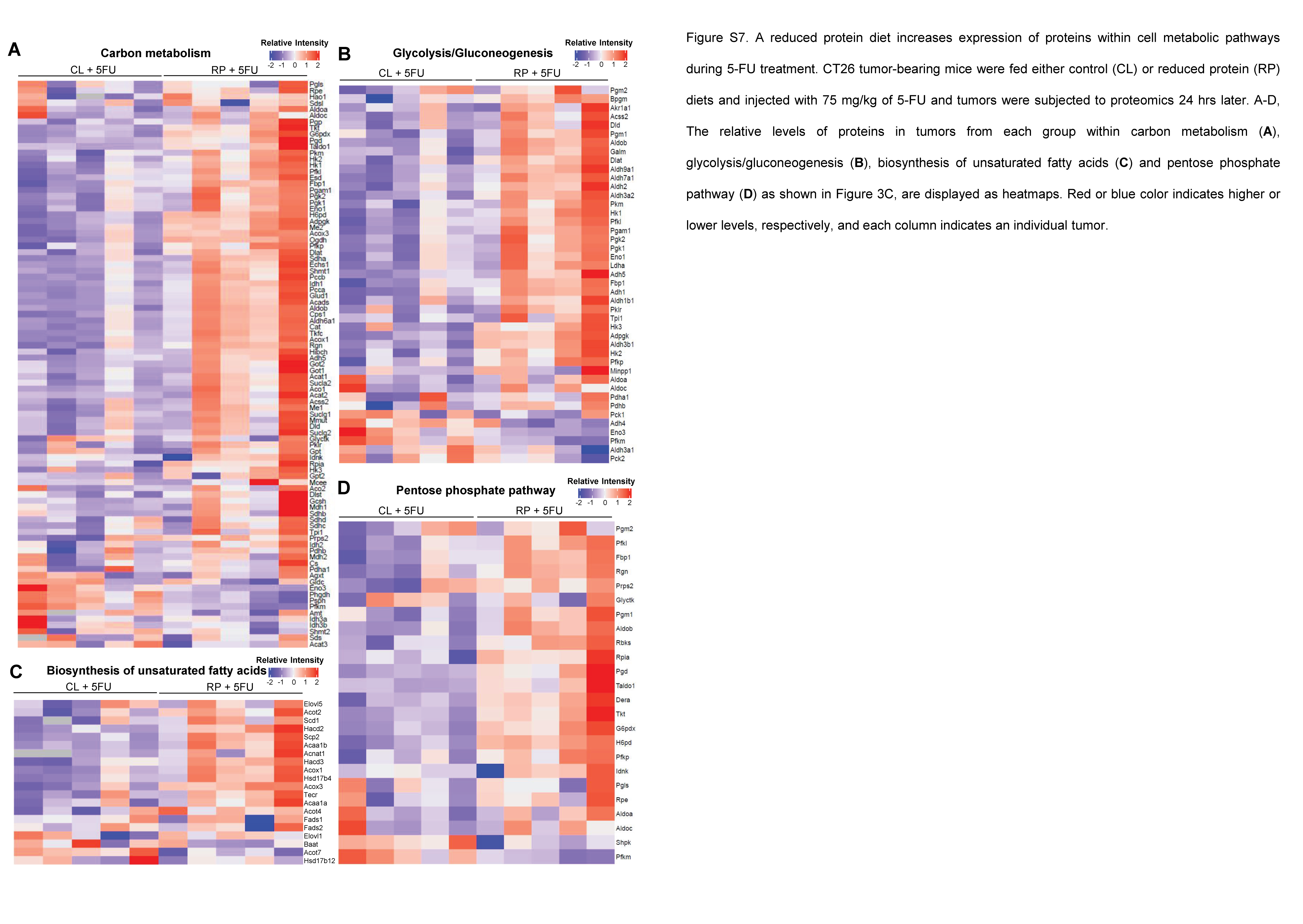

Supplement: Supplementary Information [file EMS204084-supplement-Supplementary_Information.zip › supp_info_7.tif]

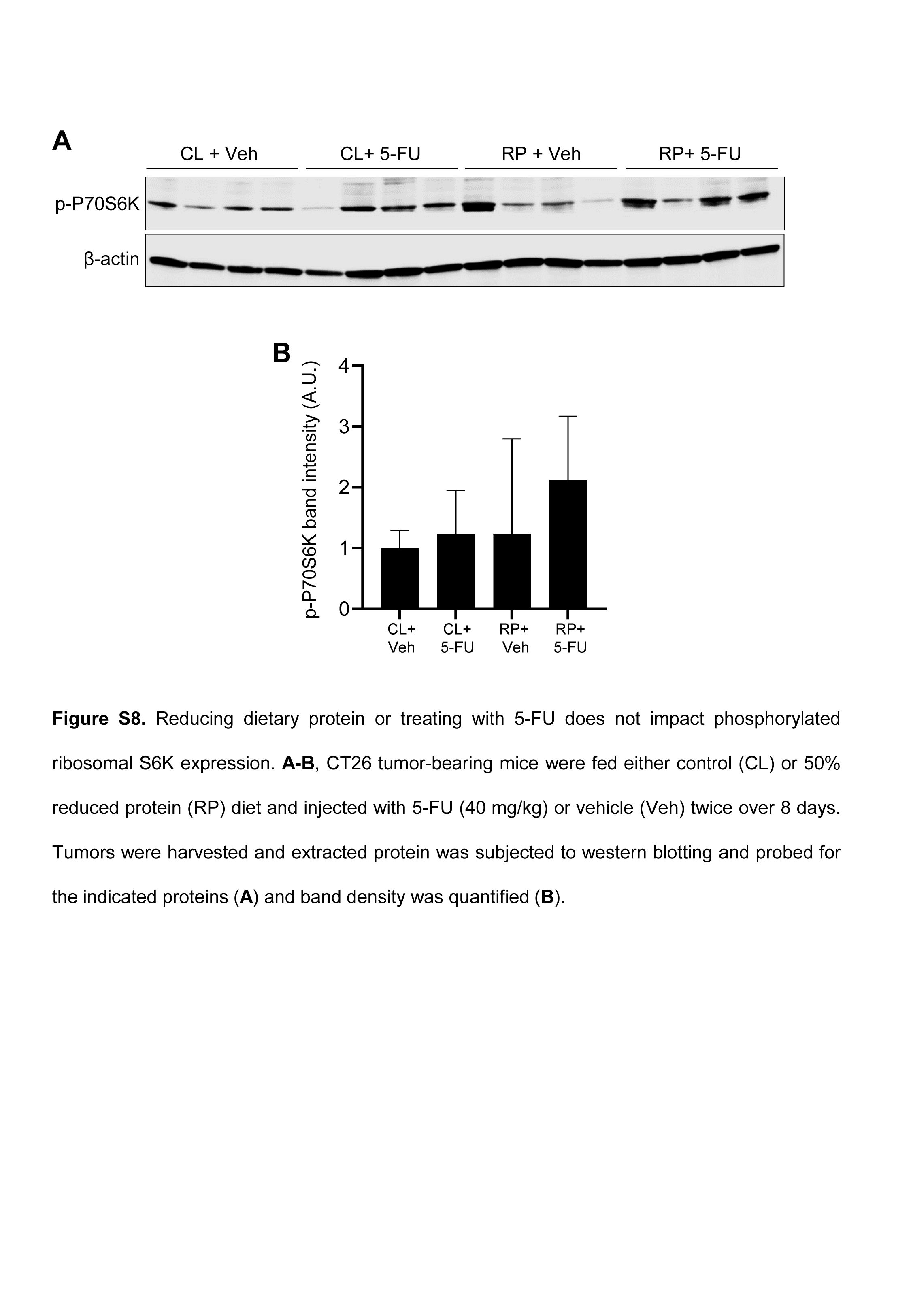

Supplement: Supplementary Information [file EMS204084-supplement-Supplementary_Information.zip › supp_info_8.tif]
